# Comparison of Consumption Data and Phenotypical Antimicrobial Resistance in *E. coli* Isolates of Human Urinary Samples and of Weaning and Fattening Pigs from Surveillance and Monitoring Systems in Germany

**DOI:** 10.3390/antibiotics11010028

**Published:** 2021-12-27

**Authors:** Octavio Mesa-Varona, Ides Boone, Matthias Flor, Tim Eckmanns, Heike Kaspar, Mirjam Grobbel, Bernd-Alois Tenhagen

**Affiliations:** 1 Department Biological Safety, German Federal Institute for Risk Assessment (BfR), 10589 Berlin, Germany; Matthias.Flor@bfr.bund.de (M.F.); Mirjam.Grobbel@bfr.bund.de (M.G.); Bernd-Alois.Tenhagen@bfr.bund.de (B.-A.T.); 2 Department for Infectious Disease Epidemiology, Robert Koch Institute (RKI), 13353 Berlin, Germany; Boone.I@rki.de (I.B.); Eckmanns.T@rki.de (T.E.); 3 Federal Office of Consumer Protection and Food Safety (BVL), Reference Laboratories, Resistance to Antibiotics Unit Monitoring of Resistance to Antibiotics, Department Method Standardization, 12277 Berlin, Germany; Heike.Kaspar@bvl.bund.de

**Keywords:** AMR, AMU, one health, clinical isolates, nonclinical isolates, weaning pig, fattening pig, *E. coli*

## Abstract

Antimicrobial resistance (AMR) data from humans are mostly collected from clinical isolates, whereas from livestock data also exist from colonizing pathogens. In Germany, livestock data are collected from clinical and nonclinical isolates. We compared resistance levels of clinical and nonclinical isolates of *Escherichia coli* from weaning and fattening pigs with clinical outpatient isolates of humans from urban and rural areas. We also studied the association of AMR with available antimicrobial use (AMU) data from humans and pigs. Differences between rural and urban isolates were minor and did not affect the comparison between human and pig isolates. We found higher resistance levels to most antimicrobials in human isolates compared to nonclinical isolates of fattening pigs. Resistance to ampicillin, however, was significantly more frequent in clinical isolates of fattening pigs and in clinical and nonclinical isolates of weaning pigs compared to isolates from humans. The opposite was observed for ciprofloxacin. Co-trimoxazole resistance proportions were higher in clinical isolates of weaning and fattening pigs as compared to isolates from humans. Resistance proportions were higher in clinical isolates than in nonclinical isolates from pigs of the same age group and were also higher in weaner than in fattening pigs. Significant associations of AMU and AMR were found for gentamicin resistance and aminoglycoside use in humans (borderline) and for ampicillin resistance in clinical isolates and penicillin use in fattening pigs. In summary, we found significant differences between isolates from all populations, requiring more detailed analyses supported by molecular data and better harmonized data on AMU and AMR.

## 1. Introduction

Antimicrobial resistance (AMR) reduces the effectiveness of antimicrobials for treating and controlling diseases and has critical economic implications [1,2]. Reducing antimicrobial use (AMU) is a main intervention to control AMR [3,4,5,6,7]. This has been shown in the human [3,4,5,8] and animal [3,4,5,9] sectors. The association of AMR with AMU cannot always be directly observed in field data. This may be due to a wealth of other factors influencing AMR [10]. Efforts are being made to reduce AMU in humans [11] and animals [12] to reduce AMR in Europe.

Surveillance and monitoring systems are part of the strategies of the Global Action Plan to control AMR [13]. These systems collect data on AMU in a country from humans and animals and observe the resistance level of zoonotic and indicator bacteria from isolates from animals, food and humans.

*Escherichia coli* are commensal bacteria from the human and animal microbiota accepted as AMR indicators [14]. These Gram-negative bacteria can, however, also cause intestinal and extraintestinal diseases in animals and humans [15]. *Escherichia coli* plays a role in transferring resistance genes between humans and animals [16,17].

In Europe, human resistance data are mostly based on clinical isolates. Resistance of nonclinical isolates is not frequently analyzed outside screening systems for specific resistant bacteria such as methicillin resistant *Staphylococcus aureus* (MRSA) [18,19].

In contrast, most published AMR data from livestock originate from nonclinical isolates. In Europe, monitoring of AMR in nonclinical isolates is harmonised by the Commission Implementing Decision (CID) 2020/1729/EU that succeeded CID 2013/652/EU. Resistance data are collected by the European Food Safety Authority (EFSA) [17]. However, there is no harmonized approach to collecting AMR-data in clinical isolates in Europe yet [20].

Prescription data from humans are provided as Defined Daily Doses (DDD). In the animal sector, sales data on AMU collected in Europe currently provide only a general overview [20]. Farm-level data are required for a more comprehensive assessment. In Germany, these data are provided as therapy frequency (TF) based on the national minimizing strategy for antibiotics in animals according to the German drug act [21].

There is an increasing awareness that AMU in livestock may influence the development of AMR in human medicine [22,23]. However, it is unknown to what extent this occurs [24]. Transmission of resistant bacteria across species may occur through food, direct contact, and common natural sources such as water [25]. However, the effect of the AMU reduction in livestock on AMR in both humans and animals is not entirely clear [26].

The adoption of a One Health approach, combining human, animal and environment sectors can help to clarify how resistance genes are transferred between reservoirs/populations. A main challenge to address this approach is to overcome the lack of harmonization in data on AMU and AMR [20].

This study compares resistance in clinical *E. coli* isolates from urinary samples from humans in urban and rural areas with resistance data on clinical and nonclinical *E. coli* isolates from weaning and fattening pigs, including antimicrobial use as explanatory factor. This work will help to clarify a possible resistance exchange of isolates between pigs and humans.

## 2. Material and Methods

### 2.1. Data Collection and Processing

Phenotypic resistance data of clinical *E. coli* isolates were collected from 2015 to 2017 from urinary samples of humans in Germany. Only outpatient isolates from urinary samples were included in the analyses. Screening isolates were excluded. Isolates were stratified as rural or urban based on the classification of the sampling site according to the German Federal Institute for Research on Building, Urban Affairs and Spatial Development (BBSR) [27]. Resistance data were extracted from the German national antibiotic resistance surveillance system (https://ars.rki.de/, accessed on 12 March 2020).

Data on clinical and nonclinical *E. coli* isolates from pigs were collected in the same period. Clinical isolates originated from various sample types and were collected by the German Resistance Monitoring of Veterinary Pathogens (GERM-VET). Nonclinical isolates from pigs originated from the German Zoonoses-Monitoring program (ZoMo). They included isolates from faecal samples of pigs at farm and caecal samples at slaughter. Isolates from pigs were stratified as from weaning pigs and fattening pigs as described by the different monitoring schemes. Isolates from pigs at slaughter were assigned to fattening pigs. Clinical isolates from pigs were available for all three years. Nonclinical isolates from fattening pigs were available for 2015 and 2017. Nonclinical isolates from weaning pigs were available for 2015 only.

Antimicrobial susceptibility was tested by broth microdilution (pig isolates) or automated test-systems (human isolates) according to the ISO 20776-1 [28,29,30,31]. Minimum Inhibitory Concentrations (MIC) were interpreted by clinical breakpoints (CBP) from the European Committee on Antimicrobial Susceptibility Testing (EUCAST) standard (Table S1).

Duplicate isolates were removed from datasets. We included the antimicrobials that had been tested in clinical isolates from both humans and pigs (i.e., ampicillin, cefotaxime, ciprofloxacin, gentamicin and co-trimoxazole) and in both clinical isolates from humans and nonclinical isolates from pigs (i.e., ampicillin, cefotaxime, ciprofloxacin, gentamicin and trimethoprim) between 2015 and 2017. Phenotypical resistance data to colistin produced with automated methods are not considered reliable [32]. Therefore, colistin had to be excluded from the analysis despite its potential relevance in a One Health context. Tetracycline was not routinely included in the test panel for human isolates and was therefore also excluded from the analysis, despite its substantial importance in pigs. None or very few isolates from pigs were resistant to ceftazidime, meropenem and tigecycline. These data were, therefore, also excluded.

Community data on AMU in humans are provided in Defined Daily Doses (DDD)/1000 inhabitants/day by the European Surveillance of Antimicrobial Consumption Network (ESAC-Net) [33]. In contrast, farm level AMU of pigs was collected as TF per semester [21]. In our study we combined the results of the two six months periods of the calendar year in the analysis. AMU data could not be analytically compared between humans and pigs.

### 2.2. Approach to Compare AMU between Population Types

To analyze the association between humans and pigs at the descriptive level, data were expressed as usage proportion of a drug class as a proportion of the total usage (proportion of relative usage) within population types (i.e., humans, weaning pigs and fattening pigs) from 2015 to 2017 applying the following formula:

PRU = (UD_2015_ + UD_2016_ + UD_2017_) × 100/(TU_2015_ + TU_2016_ + TU_2017_)

PRU: The proportion of relative usage of a drug class between 2015 and 2017 within a population. UD: Usage of a drug class within a population. TU: Total usage of all drug classes considered in a population.

The resistance proportion was defined as the number of resistant isolates divided by the total tested isolates in percentage. To be able to compare AMU and AMR, resistance proportions were also expressed relatively, i.e., as the percentage of resistance of a drug in relation to the total amount of resistances (sum of all resistance percentages) from the rest of the considered drugs (proportion of relative resistance). Proportions of relative resistance were expressed within population types (i.e., humans, weaning pigs and fattening pigs) for the studied time range by applying the following formula:

PRR = (RP_2015_ + RP_2016_ + RP_2017_) × 100/(TR_2015_ + TR_2016_ + TR_2017_)

PRR: The proportion of relative resistance to a drug in a population between 2015 and 2017. RP: Resistance proportion to a drug in a population. TR: Sum of all drug resistance proportions in a population.

### 2.3. Statistical Analysis

Logistic regression analyses were applied using the software “R” (Version 3.6.3) and “ROCR” and “pscl” R packages. The “Konstanz Information Miner (KNIME)” tool (Version 4.1.2) was used for data management and transformation. MIC values were encoded as 0/1 referring to susceptible/resistant [34].

Analyses were performed to test the following hypotheses:

- First hypotheses:
  (a) The resistance level in *E. coli* differs between urban and rural isolates of humans.
  (b) There are differences on the resistance level of *E. coli* between age groups in pigs.
  (c) The level of *E. coli* resistance is higher in clinical isolates in comparison to nonclinical isolates from pigs.
- Second hypotheses:
  (d) There are differences between the resistance levels of clinical and nonclinical isolates from weaning and fattening pigs and those from the human areas in the community.
  (e) There are similar AMR patterns between pigs and humans.
  (f) There is an association between AMU and AMR in humans and pigs.
  (g) *E. coli* resistance levels are reduced due to measures taken to control AMR.

First, resistance was compared between the isolates of the different strata within populations. This was done using univariable logistic regressions. We compared specifically the following isolates:

(a) Isolates from urban and rural human populations across years.
(b) Clinical isolates of weaning pigs and fattening pigs across years.
(c) Nonclinical isolates of weaning pigs and fattening pigs in 2015.
(d) Clinical isolates and nonclinical isolates of weaning pigs in 2015.
(e) Clinical isolates and nonclinical isolates of fattening pigs in 2015 and 2017.

Based on the results, we decided not to collapse data from different populations.

Second, we assessed the differences of resistance occurrence between isolates of each pig population (i.e., clinical/nonclinical isolates from weaning/fattening pigs) and isolates from the human community per area (i.e., urban or rural) from 2015 to 2017. Human and pig isolates were analyzed by pairwise comparison of each group within the population (i.e., isolates of humans (rural and urban) vs. isolates of pigs (clinical and nonclinical isolates of weaning and fattening pigs)). Logistic regression analyses were applied assessing the year and the human/animal independent factors.

Data on nonclinical isolates of weaning pigs were only available in 2015 and of fattening pigs in 2015 and 2017. Therefore, only data from 2015 were used to assess by univariable analyses the human/animal factor comparing clinical isolates from humans in each area and nonclinical isolates of weaning pigs. The year and the human/animal factors were only and directly assessed by multivariable analyses comparing clinical isolates from humans in each area and nonclinical isolates of fattening pigs.

We also analyzed changes in AMU and resistance occurrence in the populations over time. Analyses were performed per drug class, population type (weaning/fattening pig/ human) and isolate type (clinical/nonclinical) to assess the association of AMR with AMU and year. Where multivariable analysis was possible, AMU and year showed a high degree of correlation. Therefore, multivariable analyses were not performed. Separate AMU data for trimethoprim in pigs was not available as trimethoprim is rarely used as an individual substance in pigs as there are no veterinary medicinal products for pigs that contain trimethoprim as the sole active ingredient in Germany [35].

Further, trimethoprim, as an individual substance, was only tested in the antimicrobial panel for nonclinical isolates. In this case, an analysis was carried out only to assess changes in AMR over time between 2015 and 2017.

A *p*-value of less than 0.05 was considered statistically significant.

## 3. Results

Resistance proportions, 95% confidence intervals (CI) and the number of resistant and total number of tested *E. coli* isolates per antimicrobial of all populations are summarized in Table 1 and Table S2.

### 3.1. Comparison of Subgroups within the Population

**Figure 1:**
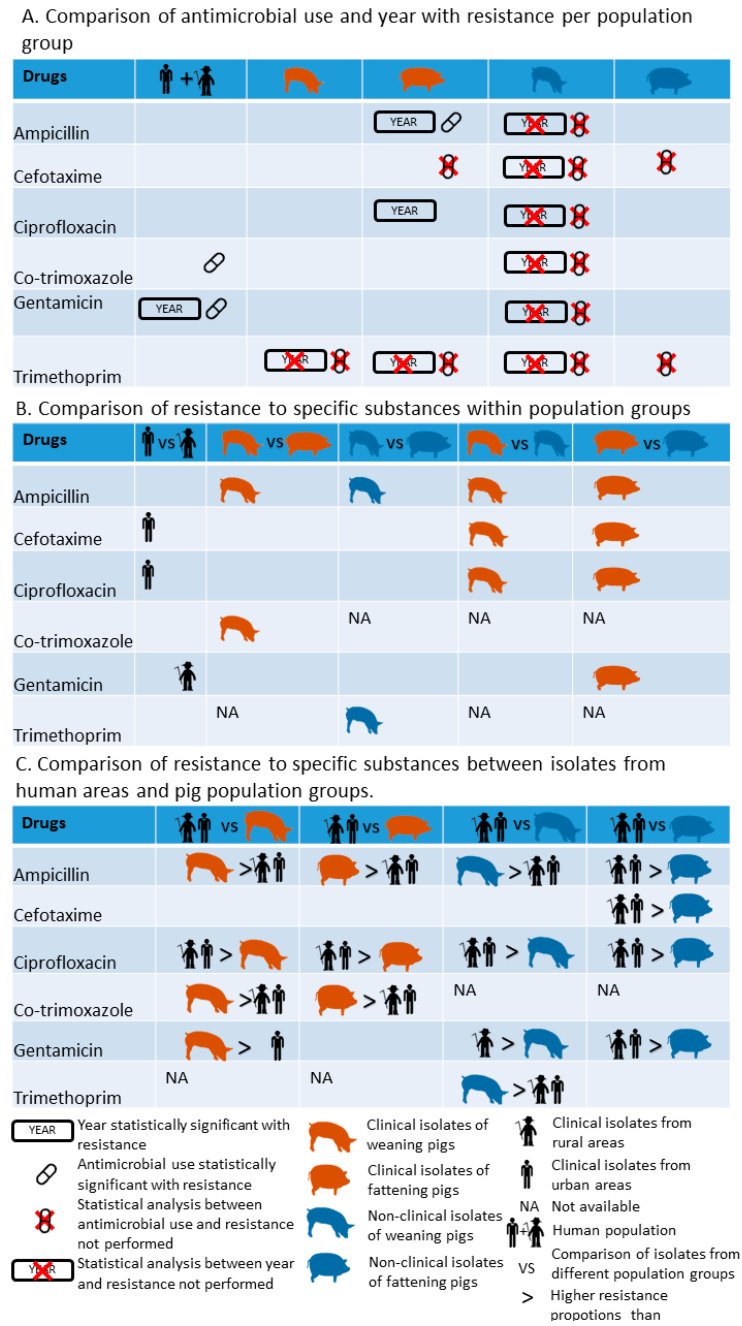
Comparison of analysis between and within populations isolates from humans and pigs.

Figure 1 shows the comparison of analysis between and within populations isolates from humans and pigs.

Resistance to gentamicin was significantly higher in human isolates from rural than from urban areas (*p* < 0.001, Table S2 and Figure 1). Resistance levels tended to be higher in urban than in rural areas for cefotaxime (*p* = 0.04) and ciprofloxacin (*p* = 0.04).

Resistance in clinical isolates from weaning and fattening pigs to the four investigated substances was always higher than in nonclinical isolates. Only resistance to gentamicin was similar between clinical and nonclinical isolates from weaning pigs (Supplementary Table S2 and Figure 1).

Resistance levels were higher in isolates from weaning pigs than from fattening pigs for ampicillin, co-trimoxazole and trimethoprim. No significant differences were found in isolates from weaning and fattening pigs for cefotaxime, ciprofloxacin and gentamicin. (Table S2 and Figure 1).

### 3.2. Comparison between Human and Pig Isolates

Higher resistance levels to ampicillin and co-trimoxazole were found in clinical isolates from weaning and fattening pigs as compared to clinical human isolates from rural and urban areas. The opposite was found for ciprofloxacin. For gentamicin, resistance in clinical isolates of weaning pigs was higher than in isolates of urban areas (Table 2, Supplementary Table S1 and Figure 1).

Resistance to ampicillin and trimethoprim in nonclinical isolates of weaning pigs was higher than in human clinical isolates from urban and rural areas. The contrary was found for ciprofloxacin. Resistance to gentamicin was lower in nonclinical isolates of weaning pigs in comparison to rural human isolates (Table 2 and Table S1 and Figure 1).

Resistance levels in nonclinical isolates of fattening pigs were lower compared to human isolates from rural and urban areas for all drugs except for trimethoprim that showed a similar resistance level (Table 3 and Table S1 and Figure 1).

### 3.3. Association of AMU and Year with Resistance within Populations

**Figure 2:**
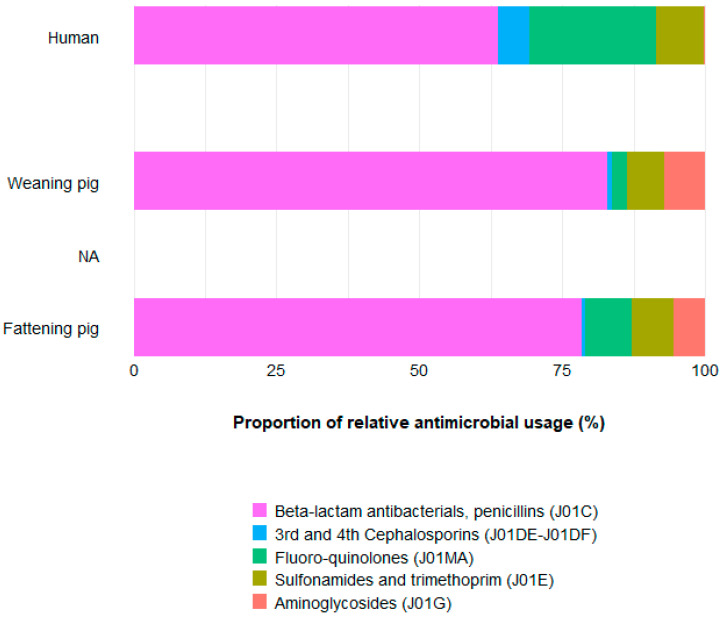
Proportion of relative use in humans, weaning and fattening pigs.

The proportion of relative use of the different drug classes differed substantially between humans and both pig groups. While level of usage in pigs contrasted between age groups, the proportion of relative use of the investigated drug classes did not differ (Table S3; Figure 2).

The proportion of relative use in humans differed substantially from those in animals. The relative use of penicillins (ATC code J01C) applied was lower in humans than in pigs. The contrary was observed for third and fourth generation cephalosporins (J01DE-J01DF) and for fluoroquinolones (J01MA). The proportions of relative use of aminoglycosides (J01G) in humans was marginal (0.2%), while it contributed a 7.2% and 5.6% of treatments in weaning and fattening pigs, respectively. Relative use of trimethoprim and sulphonamides (J01EA) was marginally higher in humans than in animals.

In line with that, AMR patterns likewise differed between humans and pigs. In humans, the proportion of relative resistance to cefotaxime and ciprofloxacin was higher than in pigs, while the relative resistance proportion to ampicillin was lower than in weaners and fattening pigs. Interestingly, the resistance proportion to gentamicin did not differ substantially, despite the substantial differences in aminoglycoside use.

**Figure 3:**
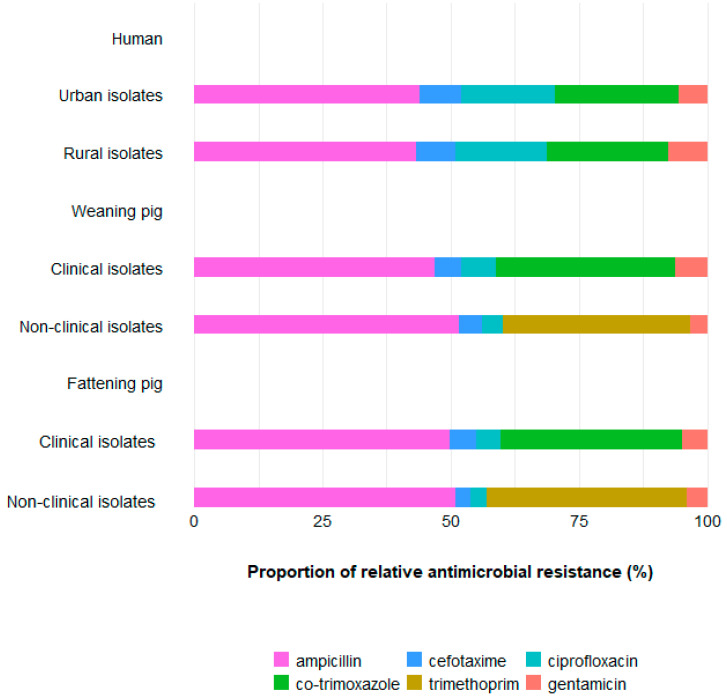
Proportion of relative resistance in all populations. In nonclinical isolates from pigs, resistance to trimethoprim was considered instead of resistance to the combination of trimethoprim and sulphonamides as data on resistance to the combination were not available.

Relative resistance proportions were similar between pig categories within isolate type. Relative resistance patterns differed between human areas and between humans and pigs in clinical isolates (Figure 3).

Positive associations between AMU and AMR were encountered in clinical isolates of fattening pigs for ampicillin and in human isolates for co-trimoxazole. This association was also found between the consumption of aminoglycosides and gentamicin in human isolates despite the fact that gentamicin is very rarely used in humans. Associations between year and AMR were found in clinical isolates of fattening pigs for ampicillin and ciprofloxacin and in human isolates for gentamicin (Table 4).

## 4. Discussion

This work compares resistance levels in clinical human outpatient isolates of *E. coli* from rural and urban areas with those in clinical and nonclinical isolates from pigs of two age groups (i.e., weaning and fattening pigs). The main objective was to see if isolates from pigs and humans would show similar resistance proportions, which might indicate a resistance exchange of isolates between humans and pigs. Exposure to isolates from pigs could occur through occupational or environmental contact (hence the stratification of urban and rural isolates) or via food. The latter might be a special issue in Germany on account of the frequent consumption of raw minced pork in some areas of the country [36].

We found significantly higher resistance levels in isolates from humans of both areas (rural and urban) to ampicillin, cefotaxime, gentamicin and ciprofloxacin than in nonclinical isolates from fattening pigs. This is noteworthy, as nonclinical isolates from fattening pigs are the isolates that are most likely to contaminate the pig carcass at slaughter and subsequently the meat. In contrast, clinical isolates from this pig group were more frequently resistant to ampicillin and co-trimoxazole than the human isolates. On the one hand, this points to substantial differences between clinical and nonclinical isolates from fattening pigs. On the other hand, it indicates that resistance in outpatient isolates differed substantially from those of fattening pigs.

The differences between clinical and nonclinical isolates from fattening pigs were confirmed by our analyses. Resistance was more frequent in clinical isolates than in nonclinical isolates in both pig categories. This is in line with the literature for pigs and calves [37,38]. A different situation was found in several EU countries comparing clinical and nonclinical isolates of *E. coli* from broilers and turkeys. In those studies, resistance proportions were higher in nonclinical isolates for several antimicrobials. The reason for these different patterns in poultry, pigs and calves remains unclear and warrants further investigation [37,39]. Higher resistance levels in clinical isolates were expected as these might be successful specific clones that have been exposed repeatedly to treatment over time. This would be in agreement with our results in the pig populations. However, this does not explain the contrary results in poultry. In cattle, it has been pointed out that isolates from different age groups in cattle show different levels of AMR and, hence, cattle at slaughter that are included in EU-wide monitoring may have lower resistance proportions than young calves. We evaded this problem in our study by considering the two age categories of pigs separately, and still we found higher resistance levels in the clinical isolates.

Ideally, nonclinical isolates from pigs should be compared to nonclinical isolates from humans. However, the latter are not routinely available. The reason for testing nonclinical isolates in the monitoring is that they are more likely to be transmitted via the food chain than clinical isolates, as only healthy animals are allowed for slaughter in the EU. As there is no straightforward and practical solution to this conflict, it needs to be considered when comparing resistance levels from animals and humans in the framework of, for example, source attribution.

With our data, we confirmed that older pigs have lower resistance proportions to ampicillin, cefotaxime and ciprofloxacin than weaner pigs, which is in line with our expectations and with differences in AMU between the age categories [21,40,41,42]. No significant difference was seen for resistance to gentamicin. In consequence, surveillance programs need to accurately define the included pig population when comparing data over time or between populations, e.g., in different countries. This standardization is well established in the harmonized monitoring according to CID 2020/1729/EU and its predecessor CID 2013/652/EU. Here pigs are sampled at slaughter providing a high degree of comparability between countries and over time. For obvious reasons, such a harmonized sampling point in time is not feasible for clinical isolates. Hence, there needs to be a thorough definition of populations if resistance is to be compared, e.g., within the same sector but in different countries.

Interestingly, nonclinical isolates from weaner pigs displayed higher resistance levels to ampicillin and trimethoprim than isolates from humans. However, exposure of humans—except for occupational contact in the farming community—to these pigs and their bacterial flora is minimal. Therefore, transmission of bacteria from these pigs to humans is unlikely.

We only used human outpatient isolates, as inpatient isolates are probably influenced by the hospital conditions [43]. Therefore, it could be assumed that outpatient isolates are more likely to directly originate from nonhuman sources than inpatient isolates. We stratified urban and rural human isolates to investigate whether resistance proportions in human isolates from rural areas were more similar to those from pigs, than isolates from urban people. People in rural areas are more likely to be exposed to either farm animals or emissions from farms or to people working on pig farms. Despite a high number of isolates considered, substantial differences in resistance proportions between human rural and urban isolates were only observed for gentamicin (higher in rural isolates), while for all other antimicrobials differences were either insignificant (ampicillin, co-trimoxazole and trimethoprim), or small (cefotaxime and ciprofloxacin slightly higher in urban areas). Moreover, resistance to gentamicin in rural areas varied over the years. In contrast to our findings, a recent study found differences in resistance levels between urban and rural areas from Great Britain in soil [44]. However, other studies did not find such differences in other parts of the world [45,46]. Work on cephalosporin-resistance in *E. coli* in different populations found different clones in the pig farming community (i.e., similar genes in the pig farming community and pig isolates) compared to the general population and attributed this to the close contact between pigs and the pig farming community [43]. However, the pig farming community is only a very small proportion of the rural population in most parts of Germany, and most people in rural areas have quite as little contact to farm animals as urban people [47]. Hence, our stratification criterion was probably too general to see differences associated with occupational exposure. Therefore, our results need to be considered carefully, as many factors may be involved, such as the type of urban/rural classification itself, differences on the livestock distribution in a country (as it is the case of Germany [48]), and differences in living conditions and hygiene in different parts of the world.

Antimicrobial use tended to decrease in all three observed populations (i.e., humans, weaning pigs and fattening pigs) for a number of substances. One major exception showing an increase in use was beta-lactam antibacterial agents in humans. Small increases were observed in the use of trimethoprim and derivatives in humans and of fluoroquinolones in pigs. The overall decrease in antimicrobial use was more pronounced in animals than in humans. This further reduces the risk of transmission of resistant bacteria from pigs to humans. In addition, the lower selective pressure could lead to a decrease in resistance to some antimicrobials in humans and pigs over the years.

We investigated the association of AMR with use in two ways. On the one hand, changes in use were compared to changes in AMR per population group. On the other hand, proportion of relative use (i.e., the proportion of a substance group use by the total use) was compared descriptively across populations and to the proportion of relative resistance (i.e., the proportion of a drug resistance by the sum of all drug resistance percentages).

To analyze the association between AMU and AMR in humans, data on AMR in human isolates from rural and urban areas were combined as no stratified use data were available. Moreover, as pointed out before, differences in AMR between rural and urban isolates were minimal. A significant positive association between use of aminoglycosides in humans and resistance to gentamicin was observed. However, use of aminoglycosides in humans was minimal and, therefore, very minor changes in use might lead to an overestimation of the effect. Substantial fluctuations in resistance to gentamicin, despite a high number of samples, further support this uncertainty. The reasons for these substantial changes in AMR are not clear.

A minor positive association (*p* = 0.045) was also seen for use of sulfonamides and trimethoprim with resistance to co-trimoxazole. However, changes in both use and resistance were small.

For the other substances, no association of use and resistance was observed. This may partly be due to the short time period and the very limited decrease in use. On an international level, a positive association of AMR with AMU in humans on country levels was observed for cephalosporins and fluoroquinolones [3,4,5]. However, neither the change in AMU of these substances nor in AMR were pronounced enough in our study to observe changes in the short time period. On the other hand, relatively higher use of these substances in humans as compared to pigs was associated with differences in AMR between human isolates and those from animals.

In pigs, a significant positive association was observed with beta-lactam use in fattening pigs and resistance to ampicillin in clinical isolates from fattening pigs; both use and resistance declined over time. As in humans, the limited length of the observation period was a likely contributor to the failure to see an effect. Over a longer period, a decrease in AMR has been seen in isolates from pigs along with a decrease in use in Germany as well as in the Netherlands [21,49].

Proportion of relative use is an approach to compare proportions of use between populations with AMU data collected in different units. The approach compares use patterns rather than actual use. The corresponding proportion of relative resistance was calculated to facilitate comparison of pattern of use ratio with pattern of resistance across population groups.

The results of this comparison highlighted that the proportion of use of, and resistance to, the highest priority critically important antimicrobials (3rd and 4th generation cephalosporins and fluoroquinolones) are higher in humans than in pigs of both age categories. Conversely, the proportion of use of penicillins and resistance to ampicillin and the proportions of use of sulphonamides and trimethoprim and resistance to co-trimoxazole or trimethoprim are higher in pigs. This may indicate that apart from the absolute level of resistance, the pattern of resistance is driven by the use pattern. However, no difference was seen in the proportion of resistance to gentamicin, while the proportion of use of aminoglycosides was much higher in pigs. This may be explained by the broad range of aminoglycosides in use that do not all select for resistance to gentamicin. Exclusion of gentamicin only marginally changed the pattern of resistance to the other substances.

To our knowledge, this is the first study analyzing statistically phenotypic resistance in clinical *E. coli* isolates from urinary samples in humans in urban and rural areas and resistance in clinical and nonclinical *E. coli* isolates from weaning and fattening pigs while considering AMU changes over time. Using a different approach, and working with national data of a number of countries from the European Economic Area (EEA), the “Analysis of antimicrobial consumption and resistance (JIACRA)” reports compare resistance levels of clinical isolates of *E. coli* from bloodstream infections and meningitis in humans and nonclinical isolates from caeca samples from fattening pigs [3,4,5]. They also studied associations of resistance to a specific substance and the use of a specific substance class in the EU countries. These were positively associated for humans as well as for animals underlining the decisive role of use for resistance development. However, the latest JIACRA report showed no association between the use of aminopenicillins and resistance to ampicillin in human *E. coli* isolates, but it did show this association in animals which is in line with our results [5]. However, we only found a significant association of penicillin-use with resistance in clinical isolates from fattening pigs.

### Limitations

This is a One-Health study that faces important limitations due to the lack of harmonization on AMU and AMR. Therefore, our results must be interpreted carefully. The panel of antimicrobials that isolates from humans and animals are tested against differs. We could only analyze the overlap between those panels. Some proposals have been made to overcome the lack of harmonization on the laboratory methods and procedures [37] and on the antimicrobial panel applied in monitoring and surveillance systems in clinical isolates of animals in Europe [50,51]. These are steps to achieve a more consistent One Health approach. Other issues remain, such as the reporting and accuracy of automated antimicrobial susceptibility testing systems that are frequently used in the human side [52]. Some, such as the VITEK 2 system, seem to provide a higher accurate assessment than others [52,53].

On the AMU side, proposals have already been provided to overcome the lack of harmonization of AMU data [20,37,54]. Regulation (EU) No. 6/2019 on veterinary products will contribute to harmonize data on antimicrobial usage in livestock in Europe. However, our study points to the necessity of clearly defining the animal populations addressed to obtain data that are comparable across countries. Moreover, issues on the comparability of exposure of animal populations have been raised using the concept of defined daily doses in the veterinary field. On account of the massive growth of young meat producing animals such as broiler chicks, a variation in time of treatment may lead to substantial over or underestimation of the number of animals actually treated [55]. It has therefore been recommended to also collect data on the number of animals treated and the dosage [20]. Further, different categories of antimicrobial classes are used for reporting AMU data in pigs and humans. In our study, AMU data were grouped according to the overlap of antimicrobial classes between the human and animal sectors, leading to a lower level of granularity. This is the case of the antimicrobial class used “Beta-lactams, penicillins (J01C)”, that includes all penicillins regardless its effect on the bacterial resistance promotion. For example, Aminopenicillins (broad spectrum penicillins) can promote resistance in both Gram-positive and Gram-negative bacteria. However, natural penicillins [56] affect only Gram-positives and do not directly select for resistance in Gram-negative bacteria as *E. coli.*

The sampling frame differs between the considered bacterial populations. While nonclinical isolates are randomly sampled, clinical isolates can only be randomized with respect to a random selection of the obtained isolates. Clinical isolates themselves are a biased population influenced by the decision of medical doctors or veterinarians to submit a sample for testing. This selection bias cannot be avoided but may be reduced by clear guidelines when to test and by the collection of sufficiently detailed clinical metadata such as information of pretreatment, severity and duration of disease and, from a One Health perspective, contact with animals. While on the one hand such data are sensitive with respect to privacy regulations; in an anonymized form they might help a lot in better understanding clinical resistance data.

Our analysis of the relative proportion of treatment and resistance has a number of limitations. A shortcoming of this approach is that a low relative proportion of use (e.g., 10%) from a group with high total AMU (e.g., 1000) may represent a much higher consumption value than a high proportion of relative use (e.g., 30%) from a group with low total antimicrobial consumption (e.g., 100). The same applies for proportions of resistance. A second shortcoming is that it does not consider cross or coresistance. For example, cephalosporins select for penicillin resistance along with resistance to cephalosporins. Resistance genes may be on the same genetic structures leading to coselection of unrelated substances. These are drawbacks that the analysis shares with other analyses on the phenotypical resistance level. Considering resistance to trimethoprim alone, instead of the combination of trimethoprim with sulphonamides, is another limitation that is specific to our analysis. However, in isolates from humans, data on resistance to co-trimoxazole and trimethoprim were available separately and differed only marginally.

## 5. Conclusions

This study found clear differences between humans and pigs with respect to the patterns of antimicrobial use and resistance of *E. coli* from the studied populations to antimicrobials. The occurrence of resistance in isolates of *E. coli* from humans and pigs was more closely associated with the level of antimicrobial usage in each population group. It was observed that the usage of drug class patterns differed substantially between humans and both pig groups. Patterns in pigs of different age categories differed among each other by the level of use but not by proportion of drug classes. Weaning pigs showed a higher antimicrobial usage than fattening pigs, in line with the differences in resistance occurrence found between both pig categories. Antimicrobials are mainly used in the diseased populations, also in concordance with the differences in resistance observed between clinical and nonclinical isolates from pigs. Minor differences between rural and urban isolates did not have an important effect when comparing isolates from pigs and humans. In most cases these isolates grouped together, showing the same trend in resistance.

In recent years, there has been a significant reduction of AMU in the human and animal sectors [12,33]. However, in the short time period considered in this study an association of change in use and in AMR was only found with respect to the resistance of clinical isolates from fattening pigs to ampicillin, which decreased along with a reduction of use of penicillins in this animal category. No divergent trends in use and resistance were observed.

A higher degree of harmonization is required on AMU and AMR data within and between sectors to conduct more in depth One-Health studies in the field.

## Figures and Tables

**Table 1:** Resistance proportions, 95% confidence intervals, number of resistant and number of tested *E. coli* isolates per antimicrobial of all populations (i.e., human areas: rural/urban; isolate type: clinical/nonclinical; pig categories: weaning/fattening) in Germany from 2015 to 2017.

	2015		2016	2017	
	Clinical	Nonclinical	Clinical	Clinical	Nonclinical
Human isolates from rural areas					
ampicillin	39.5% (37.5–41.52) 915/2317	NA	40.3% (38.2–42.43) 851/2112	39.8% (37.53–42.04) 734/1846	NA
cefotaxime	6.8% (6.14–7.58) 333/4877	NA	6.8% (6.05–7.66) 263/3862	7.1% (6.29–7.92) 274/3880	NA
ciprofloxacin	16.5% (15.69–17.41) 1207/7302	NA	16.0% (15.11–16.98) 965/6024	16.4% (15.49–17.44) 928/5645	NA
co-trimoxazole	22.3% (21.36–23.28) 1627/7295	NA	21.5% (20.5–22.59) 1296/6021	21.5% (20.47–22.64) 1215/5642	NA
gentamicin	7.1% (6.57–7.77) 521/7291	NA	8.8% (8.08–9.53) 528/6017	4.9% (4.33–5.47) 275/5646	NA
trimethoprim	22.2% (20.38–24.12) 433/1951	NA	23.3% (21.38–25.32) 423/1816	24.3%(22.08–26.59) 346/1426	NA
Human isolates from urban areas					
ampicillin	41.4% (37.84–45.06) 306/739	NA	42.2% (38.61–45.84) 313/742	39.7% (36.17–43.39) 290/730	NA
cefotaxime	7.4% (6.65–8.3) 296/3983	NA	7.0% (6.2–7.98) 233/3311	8.3% (7.4–9.27) 287/3463	NA
ciprofloxacin	17.0% (15.92–18.06) 814/4799	NA	17.0% (15.88–18.2) 696/4092	17.7% (16.52–18.91) 707/3999	NA
co-trimoxazole	22.9% (21.74–24.14) 1099/4796	NA	22.2% (20.98–23.55) 909/4088	22.0% (20.77–23.36) 881/3998	NA
gentamicin	5.7% (5.04–6.37) 272/4799	NA	5.2% (4.54–5.92) 212/4086	5.0% (4.4–5.78) 202/4003	NA
trimethoprim	23.4% (20.39–26.63) 172/736	NA	24.6% (21.54–27.91) 180/732	22.5% (18.97–26.38) 115/512	NA
Isolates of fattening pigs					
ampicillin	63.2% (54.06–71.51) 79/125	33.2% (26.95–40.02) 70/211	52.9% (45.19–60.43) 92/174	46.8% (39.17–54.54) 80/171	31.8% (27.51–36.43) 139/437
cefotaxime	5.6% (2.48–11.62) 7/125	2.4% (0.88–5.75) 5/211	5.7% (2.95–10.61) 10/174	5.8% (3.0–10.79) 10/171	1.6% (0.7–3.42) 7/437
ciprofloxacin	2.4% (0.62–7.38) 3/125	0.9% (0.16–3.75) 2/211	4.6% (2.15–9.17) 8/174	7.6% (4.28–12.92) 13/171	2.5% (1.33–4.59) 11/437
co-trimoxazole	39.2% (30.71–48.36) 49/125	NA	40.2% (32.96–47.94) 70/174	34.5% (27.52–42.2) 59/171	NA
gentamicin	6.4% (3.01–12.62) 8/125	3.3% (1.46–7.0) 7/211	5.2% (2.55–9.9) 9/174	4.7% (2.19–9.33) 8/171	2.3% (1.17–4.31) 10/437
trimethoprim	NA	26.1% (20.39–32.63) 55/211	NA	NA	24.0% (20.15–28.37) 105/437
Isolates of weaning pigs					
ampicillin	62.6% (53.38–71.03) 77/123	50.8% (44.44–57.13) 127/250	56.2% (47.72–64.28) 82/146	67.9% (59.36–75.35) 95/140	NA
cefotaxime	10.6% (5.97–17.72) 13/123	4.4% (2.33–7.95) 11/250	3.4% (1.27–8.22) 5/146	6.4% (3.17–12.21) 9/140	NA
ciprofloxacin	10.6% (5.97–17.72) 13/123	4.0% (2.05–7.46) 10/250	9.6% (5.54–15.86) 14/146	7.2% (3.7–13.18) 10/139	NA
co-trimoxazole	44.7%(35.83–53.93) 55/123	NA	44.5% (36.37–52.96) 65/146	49.6% (41.1–58.2) 69/139	NA
gentamicin	6.5% (3.06–12.82) 8/123	3.2% (1.5–6.44) 8/250	8.2% (4.51–14.23) 12/146	9.4% (5.28–15.77) 13/139	NA
trimethoprim	NA	36.0% (30.11–42.32) 90/250	NA	NA	NA

NA: Not available.

**Table 2:** Univariable logistic regression analyses between human isolates from rural and urban areas and pig populations.

Human Area	Antimicrobial	Variable	*p*-Value	OR (CI)
Clinical isolates from humans vs. clinical isolates from fattening pigs				
Rural	ampicillin	Human/Animal	<0.001	1.74 (1.44–2.1)
		Year	0.811	0.99 (0.94–1.05)
	cefotaxime	Human/Animal	0.328	0.82 (0.54–1.19)
		Year	0.698	1.02 (0.94–1.1)
	ciprofloxacin	Human/Animal	<0.001	0.27 (0.18–0.41)
		Year	0.815	0.99 (0.95–1.04)
	co-trimoxazole	Human/Animal	<0.001	2.2 (1.81–2.65)
		Year	0.329	0.98 (0.94–1.02)
	gentamicin	Human/Animal	0.159	0.75 (0.48–1.1)
		Year	<0.001	0.85 (0.79–0.91)
Urban	ampicillin	Human/Animal	<0.001	1.65 (1.35–2.01)
		Year	0.123	0.93 (0.85–1.02)
	cefotaxime	Human/Animal	0.137	0.74 (0.49–1.08)
		Year	0.199	1.06 (0.97–1.15)
	ciprofloxacin	Human/Animal	<0.001	0.26 (0.17–0.38)
		Year	0.402	1.02 (0.97–1.08)
	co-trimoxazole	Human/Animal	<0.001	2.12 (1.75–2.56)
		Year	0.358	0.98 (0.93–1.03)
	gentamicin	Human/Animal	0.989	1.0 (0.65–1.47)
		Year	0.161	0.94 (0.85–1.03)
Clinical isolates from humans vs. clinical isolates from weaning pigs				
Rural	ampicillin	Human/Animal	<0.001	2.42 (1.97–2.97)
		Year	0.431	1.02 (0.96–1.09)
	cefotaxime	Human/Animal	0.749	0.94 (0.62–1.37)
		Year	0.822	1.01 (0.93–1.1)
	ciprofloxacin	Human/Animal	<0.001	0.5 (0.35–0.69)
		Year	0.736	0.99 (0.95–1.04)
	co-trimoxazole	Human/Animal	<0.001	3.05 (2.51–3.71)
		Year	0.488	0.99 (0.95–1.03)
	gentamicin	Human/Animal	0.446	1.15 (0.79–1.62)
		Year	<0.001	0.86 (0.8–0.92)
Urban	ampicillin	Human/Animal	<0.001	2.29 (1.85–2.85)
		Year	0.975	1.0 (0.91–1.1)
	cefotaxime	Human/Animal	0.407	0.85 (0.56–1.23)
		Year	0.262	1.05 (0.96–1.14)
	ciprofloxacin	Human/Animal	<0.001	0.47 (0.33–0.65)
		Year	0.48	1.02 (0.97–1.08)
	co-trimoxazole	Human/Animal	<0.001	2.95 (2.42–3.59)
		Year	0.556	0.99 (0.94–1.03)
	gentamicin	Human/Animal	0.021	1.54 (1.05–2.18)
		Year	0.304	0.95 (0.87–1.04)
Clinical isolates from humans vs. nonclinical isolates from weaning pigs ^1^				
Rural	ampicillin	Human/Animal	<0.001	1.58 (1.22–2.06)
	cefotaxime	Human/Animal	0.138	0.63 (0.32–1.11)
	ciprofloxacin	Human/Animal	<0.001	0.21 (0.1–0.38)
	gentamicin	Human/Animal	0.02	0.43 (0.19–0.82)
	trimethoprim	Human/Animal	<0.001	1.97 (1.49–2.6)
Urban	ampicillin	Human/Animal	0.01	1.46 (1.1–1.95)
	cefotaxime	Human/Animal	0.077	0.57 (0.29–1.01)
	ciprofloxacin	Human/Animal	<0.001	0.2 (0.1–0.37)
	gentamicin	Human/Animal	0.101	0.55 (0.25–1.05)
	trimethoprim	Human/Animal	<0.001	1.84 (1.35–2.51)

An odds ratio (OR) >1 indicates a higher occurrence of resistance in isolates from the pig category and a positive association between year and AMR, i.e., an increase of AMR over time. ^1^ Data on nonclinical isolates from weaning pigs were only available for 2015. Therefore, only 2015 data from humans and pigs were used in these analyses.

**Table 3:** Multivariable logistic regression analyses between human isolates from rural and urban areas and pig populations.

Human Area	Antimicrobial	Variable	*p*-Value	OR (CI)
Clinical isolates from humans vs. nonclinical isolates from fattening pigs ^1^				
Rural	ampicillin	Human/Animal	<0.001	0.72 (0.6–0.85)
		Year	0.934	1.0 (0.95–1.06)
	cefotaxime	Human/Animal	<0.001	0.25 (0.13–0.43)
		Year	0.751	1.01 (0.93–1.1)
	ciprofloxacin	Human/Animal	<0.001	0.1 (0.06–0.17)
		Year	0.928	1.0 (0.95–1.05)
	gentamicin	Human/Animal	<0.001	0.38 (0.23–0.6)
		Year	<0.001	0.85 (0.8–0.91)
	trimethoprim	Human/Animal	0.5	1.07 (0.88–1.29)
		Year	0.277	1.04 (0.97–1.12)
Urban	ampicillin	Human/Animal	<0.001	0.69 (0.57–0.83)
		Year	0.46	0.97 (0.88–1.06)
	cefotaxime	Human/Animal	<0.001	0.22 (0.12–0.38)
		Year	0.22	1.05 (0.97–1.15)
	ciprofloxacin	Human/Animal	<0.001	0.1 (0.05–0.16)
		Year	0.323	1.03 (0.97–1.09)
	gentamicin	Human/Animal	0.005	0.49 (0.29–0.78)
		Year	0.152	0.93 (0.85–1.03)
	trimethoprim	Human/Animal	0.492	1.08 (0.87–1.33)
		Year	0.581	0.97 (0.87–1.08)

An odds ratio (OR) >1 indicates a higher occurrence of resistance in isolates from the pig category and a positive association between year and AMR, i.e., an increase of AMR over time. ^1^ Data on nonclinical isolates from fattening pigs were only available in 2015 and 2017. Only multivariable analyses were performed to compare the data of the available years.

**Table 4:** Association of year and antimicrobial use with AMR to different antimicrobials in the populations. Results of univariable logistic regression analyses.

	AMU		Year	
Antimicrobial	*p*-Value	Odds Ratio (95% CI)	*p*-Value	Odds Ratio (95% CI)
Clinical isolates from humans				
ampicillin	0.71	0.97 (0.81–1.16)	0.788	0.99 (0.95–1.04)
cefotaxime	0.311	0.52 (0.15–1.84)	0.346	1.03 (0.97–1.08)
ciprofloxacin	0.259	0.83 (0.61–1.15)	0.319	1.02 (0.98–1.05)
co-trimoxazole	0.045	189.62 (1.12–3.1 × 10^4^)	0.103	0.98 (0.95–1.01)
gentamicin	<0.001	1.09 × 10^185^ (3.8 × 10^95^–3.5 × 10^274^)	<0.001	0.88 (0.84–0.93)
trimethoprim	0.336	8.4 × 10^20^ (1.6 × 10^−22^–2.6 × 10^63^)	0.288	1.04 (0.97–1.11)
Clinical isolates from fattening pig				
ampicillin	0.006	2.22 (1.27–3.92)	0.006	0.72 (0.57–0.91)
cefotaxime	NA	NA	0.928	1.02 (0.63–1.69)
ciprofloxacin	0.122	9.3 × 10^−15^ (7.9 × 10^−36^–51.31)	0.046	1.8 (1.03–3.32)
co-trimoxazole	0.502	1.89 (0.29–12.16)	0.37	0.9 (0.71–1.14)
gentamicin	0.51	16.0 (3.1 × 10^−3^–5.45 × 10^4^)	0.524	0.85 (0.51–1.42)
Clinical isolates from weaning pig				
ampicillin	0.898	0.99 (0.91–1.09)	0.344	1.13 (0.88–1.45)
cefotaxime	0.137	2.06 × 106 (0.01–5.85 × 10^14^)	0.206	0.73 (0.44–1.19)
ciprofloxacin	0.328	2.5 × 10–4 (7 × 10^−12^–2.2 × 10^3^)	0.339	0.81 (0.53–1.24)
co-trimoxazole	0.38	0.73 (0.35–1.48)	0.414	1.11 (0.87–1.41)
gentamicin	0.436	0.56 (0.12–2.23)	0.402	1.21 (0.78–1.92)
Nonclinical isolates from fattening pig				
ampicillin	0.727	1.08 (0.7–1.64)	0.727	0.97 (0.81–1.16)
cefotaxime	NA	NA	0.5	0.82 (0.46–1.51)
ciprofloxacin	0.199	4.3 × 10^−15^ (3.4 × 10^−42^–6.78 × 10^4^)	0.199	1.64 (0.85–4.19)
gentamicin	0.445	24.12 (4.6 × 10^−3^–7.9 × 10^4^)	0.445	0.83 (0.51–1.38)
trimethoprim	NA	NA	0.573	0.95 (0.79–1.15)

An odds ratio (OR) >1 indicates a positive association of year and AMU with AMR. The association of AMU with AMR could not be assessed for cephalosporins in nonclinical isolates from fattening pigs as AMU figures remained constant. NA: Not available.

## Data Availability

Data from databases that are not openly available are published in the Zenodo platform (https://zenodo.org/record/5752387 (accessed on 3 December 2021)).

## Supplementary Materials

The following supporting information can be downloaded at: https://www.mdpi.com/article/10.3390/antibiotics11010028/s1. Table S1: Clinical breakpoints from EUCAST (1 April 2021). Table S2: Overview of the prevalence and analytical comparison between populations. Table S3: Antimicrobial usage in Germany expressed as defined daily doses (DDD/100,000inhabitants/day) in the human community and as therapy frequency per 100 animal years of weaning and fattening pigs from 2015 to 2017.
