# Supplementary material for: Comparison of Consumption Data and Phenotypical Antimicrobial Resistance in E. coli Isolates of Human Urinary Samples and of Weaning and Fattening Pigs from Surveillance and Monitoring Systems in Germany
Source: Antibiotics (Basel). 2021 Dec 27;11(1):28. doi: 10.3390/antibiotics11010028 (PMC8772873; doi:10.3390/antibiotics11010028)
Supplement: Supplementary file 1 [file antibiotics-11-00028-s001.zip › Supplementary tables_S1; S3_.pdf]

Table S1. Clinical breakpoints from EUCAST (01.04.2021).

**Antimicrobial drug tested**	**Clinical breakpoints applied**
ampicillin	>8
cefotaxime	>2
ciprofloxacin	>0.5
co-trimoxazole	>4
gentamicin	>2
trimethoprim	>4

Table S3. Antimicrobial usage in Germany expressed as defined daily doses (DDD/100,000 inhabitants/day) in the human community (1) and as therapy frequency per 100 animal years of weaning and fattening pigs from 2015 to 2017(2). (3)

	**2015**	**2016**	**2017**	**2015**	**2016**	**2017**	**2015**	**2016**	**2017**
Antibiotic class	Human			Weaning pig			Fattening pig		
Aminoglycosides (J01G)	1.43	1.4	1.38	164	108	106	24	16	12
Beta-lactams, penicillins (J01C)	330	342	382	1774	1331	1263	289	230	206
3rd and 4th Cephalosporins (J01DD-J01DE)	33.2	30.7	24.6	16	13	11	2	2	2
Fluoro-quinolones (J01MA)	132	124	111	44	45	49	27	24	24
Sulfonamides and trimethoprim (J01E)	47	46	46	148	122	81	37	19	12
Trimethoprim und derivades (J01EA)	4.94	4.99	5.08	NA	NA	NA	NA	NA	NA

[1] European Centre for Disease Prevention and Control (ECDC). Antimicrobial consumption database (ESAC-Net).

[2] Matthias Flor, A.K., Heike Kaspar, Bernd-Alois Tenhagen, Jürgen Wallmann. Arbeitsgruppe Antibiotikaresistenz des Bundesinstituts für Risikobewertung und des Bundesamtes für Verbraucherschutz und Lebensmittelsicherheit. Themenkomplex 1: Entwicklung der Antibiotikaabgabe- und -verbrauchsmengen sowie der Therapiehäufigkeit.; 2019.

[3] Values were inflated by 100 as compared to standard values to minimise decimals.
